# Supplementary material for: Apparatus for quantum-mixture research in microgravity
Source: Nat Commun. 2026 Jul 28;17:7573. doi: 10.1038/s41467-026-75968-9 (PMC13415515; doi:10.1038/s41467-026-75968-9)
Supplement: Supplementary file 1 — Transparent Peer Review File [file 41467_2026_75968_MOESM1_ESM.pdf]

nature portfolio

Peer Review File

Apparatus for quantum-mixture research in microgravity

Corresponding Author: Dr Baptist Piest

This file contains all reviewer reports in order by version, followed by all author rebuttals in order by version.

Version 0:

Reviewer comments:

Reviewer #1

(Remarks to the Author)
The manuscript presents a technically sophisticated study on the generation and release dynamics of ultracold mixtures of K41 and Rb87 using an integrated, transportable atom chip setup, —an experiment that truly stands out for its complexity. The authors provide a thorough analysis of the effects of transient magnetic fields during trap switch-off and demonstrate that an optimized switch-off protocol can effectively minimize unwanted differential release velocities between the two Bose-Einstein condensates. The experimental work is complemented by comprehensive simulations of the mixture dynamics, which show strong agreement with the experimental data for two out of three orientations of the chamber relative to gravity. This reflects both the methodological rigor and the depth of the analysis.

Having said that, the scope and novelty of the work are somewhat limited with respect to the standards of this high-impact journal. The manuscript does not describe new break-through methods nor new physics. Despite the clear applications to future space-borne applications, the present study essentially reports on an optimization of release dynamics performed on Earth, albeit for three different orientations of the setup. The main result is that transient magnetic fields during switch-off can be modeled and partially compensated to improve the reproducibility of the release. While this is certainly an important step for the optimal operation of the authors' own experimental platform, it is not of general interest to the broader ultracold atoms or quantum technology community, since similar effects have already been discussed in similar contexts, e.g. in references 29-31 of this manuscript.
In summary, this is a very well-executed, technical study of a highly complex experimental system, which is well-written with great attention to detail in both experiment and modeling. However, we feel its primary significance lies in providing an important calibration for the authors' apparatus rather than delivering broadly novel physics insight. For that reason, we are not convinced that the manuscript meets the high threshold of impact expected for this journal.

We do recognize the manuscripts quality and potential value in a more specialized venue.

====================
More detailed Comments:
====================

P3 end of 1st paragraph: "These results demonstrate the potential of atom chip setups for field operation as well as for answering fundamental questions of quantum gas physics". This sentence is slightly misleading as the paper does not demonstrate "the potential of atom chip setups […] for answering fundamental questions of quantum gas physics [33]" It is just that the cited paper suggests but any such set up would be suitable for this. Please reformulate.
P5 beginning of 1st paragraph: The temperature should be quoted with the corresponding atom numbers, since atom number and temperature tend to be anti-proportional.
P5 middle of 1st paragraph: The readability would be improved by the authors also stating the size of the CMOT (even though of course it can be calculated back from the trapping frequencies).
P5 middle of 1st paragraph: to the non-specialist reader it might be seen as somewhat unconventional that the author should use microwave evaporation rather than RF. They also should add a small comment as to why this is necessary.
P7 end of 1st paragraph: "It surpasses the performance of other mobile and compact setups by an order of magnitude" This is a very bold statement, which needs citations to support it.
P7 end of last paragraph: "It follows that, if the measured relative position between the BECs Δz is zero after a fixed time of flight, the associated relative velocity has to be zero as well." This is obviously not true in the generality stated here. A single

point coincidence of two trajectories might just indicate a crossing of the two rather than 100% overlap. The authors should clarify the assumptions behind their statement.
P9: Last paragraph: “decaying magnetic field, which mainly acts perpendicular to the atom chip surface” The authors should elaborate why the fields are perpendicular to the surface. It seems to us that some of the physics of an atom chip relies on the fact that this is not the case (e.g. IP trap).
P11 second paragraph: The vertical line is derived from a single position measurement. This seems risky, since residual magnetic gradients can push the atoms towards one side, thus making them fly in an arc. Similar effects would be expected from an initial velocity. A time-of-flight measurement of multiple points would avoid such a source of error.
P11 last paragraph: “Given the magnitude of the different systematic effects, we attribute the observed deviations in the 0.0◦ case primarily to an oversimplified release model” This statement is troubling. In a paper, which focusses on the magnetic release, we would have expected at least some level of analysis of the systematic errors. If one direction is wrong the mere coincidence of the other two directions with the prediction does not indicate that these are right. The authors should elaborate, why they expect the ‘systematic’ errors only to show up in the zero-degree case.
P12-13: Even though conclusions are perfectly stated, a final section with the conclusions might be missing at the end of the manuscript.
Methods, p14, figure 6: Please use physically meaningful parameters. The current in the coils are of no interest to the reader. Trapping frequencies and positions would be much more useful. Similarly, the absolute microwave frequency is of little consequence other than its detuning from the trap bottom.
6: Code availability: It is strange that the code should be available only under the condition of “reasonable request”. What would be an “unreasonable request”?

(Remarks on code availability)
I cannot find any code available under the URL above.

Reviewer #2

(Remarks to the Author)
I co-reviewed this manuscript with one of the reviewers who provided the listed reports. This is part of the Nature Communications initiative to facilitate training in peer review and to provide appropriate recognition for Early Career Researchers who co-review manuscripts.

(Remarks on code availability)
It redirects to: Efficient numerical description of the dynamics of interacting multispecies quantum gases. Annie Pichery, Matthias Meister et al. AVS Quantum Science, 5, 4, 12 2023.

I could not find any code to download, but the authors wrote that "The data that support the findings of this study are available from the corresponding author upon reasonable request".

Reviewer #3

(Remarks to the Author)
The paper by PIEST et al. reports on K-Rb experiments on an atom chip. The experiments are carried out in the vicinity of the MAIUS-B sounding rocket payload with the prospect to be used for experiments in microgravity. However all here reported experiments have been carried out on ground, some of them were not carried out in the payload.

The main results of this work are an improvement in atom number for both species in comparison to previous chip-experiments, an optimised release method that leads to a minimum velocity difference between the two species, studies of the overlap of the two species with respect to different angles to gravity.

The described improvements are of a pure technical nature, the reported steps are incremental and largely based on standard techniques. It is not clear, why this could be seen as a scientific breakthrough.

While the work here does exceed the atomic loading rate of previous demonstrations [11,12], it does so in a ground-based setting and not under microgravity conditions, making the result somewhat less interesting to a broad audience.

Fig. 2 seems relatively standard - what does one learn from it? Fig. 3 compares the achieved atom number and loading rate to works in literature. It is not clear why these specific references have been chosen and this should be explained better, e.g. PRL, 134, 093401 (2025) achieves a similar atom number (2x10^5 and 1.6x10^5 for Rb and K).

Fig. 4 shows profiles of mixed BECs after switch-off of the magnetic field in different orientations to Earth’s magnetic field. The experimental data seems fairly similar for both switch-off scenarios. However experiment and theory match less well for the fast switch-off. Could the authors comment on that?

From a technical point of view, the described work is interesting and will advance the field, the procedures are thoroughly and clearly explained and the results reported in detail. However, it is difficult to discern any major change of principle

compared to previous approaches.

One of the main claims and points of interest in the manuscript is that the highest flux rate for a dual Rb-K BEC chip-system was achieved. It is not clear from the manuscript why the flux rate should exceed those previously reported and whether there is a fundamental change in the approach that leads to the improvement.

In conclusion, the reported work is a relevant experimental step forwards in dual-species BEC production for space-borne experiments, but the lack of a full experimental demonstration under intended operational conditions (i.e. in microgravity) means that it is unlikely to be of the highest interest to all of Nature Communications' broad audience.

Minor issues:

•Figure 2: the font size is rather small in places, especially on the axis scales, and should be increased.

•Page 5, last paragraph: the authors quote trapping frequencies for a cylindrical trap, it should be clarified whether they are radial, axial or mean.

•Most of the manuscript is well written, but in a few places the clarity could be improved. Page 9: "magnetic field prober" -> probe. Page 6 final paragraph: “For the mixture generation, only the final frequency of the last ramp was increased by (70±10) kHz compared to the 87Rb-only case. We account this for the few µm-shift due to the repulsion with cold 41K atoms leading to an energy shift of the 87Rb atoms.” The use of “account” in this way is not, in my opinion, completely correct and the whole pair of sentences is quite clumsily structured, making it harder to read and correctly interpret.

•Figure 4 and discussion at top of page 9: the authors compare a theoretical model of atomic launching against experimental data. They state that “The model successfully reproduces the observed data…” but the reduced chi-squared value of 3.3 that is quoted in the caption of figure 4 is relatively high. This would suggest that either the model is incomplete or the experimental uncertainty has been underestimated. The authors should comment on what factors could be leading to the discrepancy.

•Figure 4 (b): “u” should be replaced with correct character “\mu” in legend.

(Remarks on code availability)

Reviewer #4

(Remarks to the Author)
I co-reviewed this manuscript with one of the reviewers who provided the listed reports. This is part of the Nature Communications initiative to facilitate training in peer review and to provide appropriate recognition for Early Career Researchers who co-review manuscripts.

(Remarks on code availability)

Reviewer #5

(Remarks to the Author)
In this manuscript, the authors describe the performance of an apparatus specifically designed for fundamental physics tests to be conducted under microgravity conditions using ultracold mixtures of Rubidium and Potassium gases. Following a brief technical introduction—highlighting in particular the high atomic flux achieved through atom chip technology—the authors report on their efforts to minimize the differential velocity between the two atomic species caused by magnetic field transients during release from the magnetic trap. The final part of the paper is devoted to the study of interspecies repulsion in the presence of the Earth's gravitational field. This study is made especially interesting by the capability of rotating the apparatus with respect to the gravitational acceleration.

The manuscript is clearly written, and the information is presented in a comprehensive and well-structured manner. Regarding the content, the topics addressed are of crucial importance in view of future space missions aimed at performing fundamental physics tests with ultracold atoms. Overall, the technology and experimental solutions presented represent the current state of the art in the field. Therefore, despite the highly technical nature of the paper, I strongly recommend its publication in Nature Communications.

I have no major comments, only a few minor remarks:

-Figure 3: The references appear to be incorrect. For example, the reference to BECCAL is [17] but should be [16], and STE-QUEST is [7] instead of [6]. In particular, reference [12] seems not directly relevant, as it refers to an optical single-species BEC (see https://journals.aps.org/prl/abstract/10.1103/PhysRevLett.123.240402

- Figure 6: To provide a more complete overview of the experimental sequence, I suggest adding the mesoscopic U and H

structures below the atom chip.

- To reduce the velocity difference, the authors chose to adjust the switch-off timing between the bias coils and the atom chip. Are there physical motivations underlying this choice, or is it primarily a heuristic approach? Could the possibility of switching off the Z-base and Z-science structures at different times also play a role?

- I recommend enlarging the font size of the plots in Figures 2, 4b, and especially Figure 5, as they are difficult to read when printed on A4 paper.

(Remarks on code availability)

Version 1:

Reviewer comments:

Reviewer #1

(Remarks to the Author)
The authors have substantially improved the manuscript. However, the main criticism remains: this is a rather technical paper about a incremental improvement of an experimental apparatus. No new physics and no substantially new techniques are presented. This manuscript still falls well short of the high standard of Nature Communications.

(Remarks on code availability)

Reviewer #2

(Remarks to the Author)
I co-reviewed this manuscript with one of the reviewers who provided the listed reports. This is part of the Nature Communications initiative to facilitate training in peer review and to provide appropriate recognition for Early Career Researchers who co-review manuscripts.

(Remarks on code availability)

Reviewer #4

(Remarks to the Author)
I co-reviewed this manuscript with one of the reviewers who provided the listed reports. This is part of the Nature Communications initiative to facilitate training in peer review and to provide appropriate recognition for Early Career Researchers who co-review manuscripts.

(Remarks on code availability)

Reviewer #5

(Remarks to the Author)
In this new version of the paper, the authors have significantly extended their work from both an experimental standpoint, adding a microgravity study conducted using an Einstein Elevator, and a theoretical one, providing a compelling interpretation of the discrepancy between simulations and experimental data regarding the 0° release of the condensates.

I have no specific comments and, as far as I am concerned, the paper can be published in its current form without further revisions.

Regarding the concerns raised by other referees about the technical nature of the paper, I believe the addition of the microgravity experiment is sufficient to make the manuscript suitable for a broader readership, as the authors themselves point out. The demonstrated ability to accurately control the position and velocity of atomic samples is of crucial importance for future fundamental physics experiments in space; given the significant interest in this field, it cannot be dismissed as a mere technical detail.

(Remarks on code availability)

Open Access This Peer Review File is licensed under a Creative Commons Attribution 4.0 International License, which permits use, sharing, adaptation, distribution and reproduction in any medium or format, as long as you give appropriate credit to the original author(s) and the source, provide a link to the Creative Commons license, and indicate if changes were made.
In cases where reviewers are anonymous, credit should be given to 'Anonymous Referee' and the source.
The images or other third party material in this Peer Review File are included in the article's Creative Commons license, unless indicated otherwise in a credit line to the material. If material is not included in the article's Creative Commons license and your intended use is not permitted by statutory regulation or exceeds the permitted use, you will need to obtain permission directly from the copyright holder.
To view a copy of this license, visit https://creativecommons.org/licenses/by/4.0/

Response to Review of „Apparatus for quantum-mixture research in microgravity"

We sincerely thank all reviewers for reading and reviewing our manuscript and for their suggested improvements. We think that they have raised important points which have led us to revise significant parts of the article. We hope that the new version of the article is now well suited for a broader readership. We will go through the comments step by step.

To address the questions of the referees, we conducted novel microgravity experiments in the Einstein-Elevator facility of Leibniz University Hannover. We believe these efforts address these questions, consolidate our conclusions, and significantly enhance the impact of the paper.

Additional remarks/changes not covered in the point-by-point answers below:

- We have updated the author list and author contribution which reflect the additional content of the revised version. One author was added, and the affiliation of one of the authors has been corrected.
- We slightly modified the ROI displayed in Fig. 5 and corrected a wrong pixel-size (factor between pixel number and µm in the data display).
- We replaced and updated Fig. 4 a,b) to reflect the new combined gravity and microgravity measurements. These measurements were performed with an updated setup after exchanging our chip and coil current drivers which explains the different fit results for the time constants. Fig. 5a-c was obtained with the previous setup; accordingly, the simulations shown there are based on the earlier decay constants.
- Fig 5d and 5h are added and show microgravity results.
- We added in the methods section some more details for the simulation of Fig 4b.

REVIEWER COMMENTS

Reviewer #1 (Remarks to the Author):

The manuscript presents a technically sophisticated study on the generation and release dynamics of ultracold mixtures of K41 and Rb87 using an integrated, transportable atom chip setup, —an experiment that truly stands out for its complexity. The authors provide a thorough analysis of the effects of transient magnetic fields during trap switch-off and demonstrate that an optimized switch-off protocol can effectively minimize unwanted differential release velocities between the two Bose-Einstein condensates. The experimental work is complemented by comprehensive simulations of the mixture dynamics, which show strong agreement with the experimental data for two out of three orientations of the chamber relative to gravity. This reflects both the methodological rigor and the depth of the analysis.

Having said that, the scope and novelty of the work are somewhat limited with respect to the standards of this high-impact journal. The manuscript does not describe new break-through methods nor new physics. Despite the clear applications to future space-borne applications, the present study essentially reports on an optimization of release dynamics performed on Earth, albeit for three different orientations of the setup. The main result is that transient magnetic fields during switch-off can be modeled and partially compensated to improve the reproducibility of the release. While this is certainly an important step for the optimal operation of the authors' own experimental platform, it is

not of general interest to the broader ultracold atoms or quantum technology community, since similar effects have already been discussed in similar contexts, e.g. in references 29-31 of this manuscript.
In summary, this is a very well-executed, technical study of a highly complex experimental system, which is well-written with great attention to detail in both experiment and modeling. However, we feel its primary significance lies in providing an important calibration for the authors' apparatus rather than delivering broadly novel physics insight. For that reason, we are not convinced that the manuscript meets the high threshold of impact expected for this journal.

We do recognize the manuscripts quality and potential value in a more specialized venue.

We thank the reviewer for the clear assessment of the manuscript and the very helpful suggestions to make this paper more impactful and interesting for a broader readership. We respectfully disagree with the referee's assessment that our work is merely a calibration of a complex device. In fact, this dual-BEC experiment outperforms by more than an order of magnitude its counterparts in terms of production flux of the mixture (see fig. 3), opening radically new perspectives for many-body physics ranging from the study of quantum droplets, phase transitions, miscibility/immiscibility physics, especially when combined with microgravity conditions. The understanding of the perfect release and control kinematics makes it in addition very suitable for field metrology and fundamental tests as promoted in the text. Furthermore, we have decided to provide a major update of the manuscript. In this new version, we have added new data which we have collected in a recent measurement campaign conducted in the microgravity environment of the Hannover Einstein Elevator. This new data shows that the apparatus is able to generate BEC mixtures with so far unmatched atom numbers in microgravity. It further shows that our simulation provides consistent results not only for different orientations in gravity but also in microgravity. We show with the new microgravity data that the described switch-off technique reduces the center-of-mass motion to zero leaving only the inter- and intra-atomic interactions that govern the BEC dynamics. We also replaced Fig. 4 which presented ground-based data and a microgravity prediction with a new figure that also adds actual microgravity data.

We believe that these new results will significantly increase the importance of the paper and make it more interesting to a broader readership. To integrate the new data into the manuscript we have merged the new result into Figure 5. We also had to change the abstract of the paper and larger parts of section 2.3 to appropriately discuss the data.

====================
More detailed Comments:
====================

P3 end of 1st paragraph: "These results demonstrate the potential of atom chip setups for field operation as well as for answering fundamental questions of quantum gas physics". This sentence is slightly misleading as the paper does not demonstrate "the potential of atom chip setups [...] for answering fundamental questions of quantum gas physics [33]" It is just that the cited paper suggests but any such set up would be suitable for this. Please reformulate.

While we are not answering fundamental physics questions in the paper, we want to stress that our setup indeed allows to conduct such studies, on ground but especially in space: While a high flux is relevant for metrological studies (like tests of the equivalence principle) and allows to average down faster, the controllability of the release allows to conduct quantum gas mixture experiments requiring

collocation, e.g. expanding atomic shells or molecular association. Both aspects have not yet been shown with atom chip-based setups. We therefore reformulate the statement to be more precise:

"These results demonstrate the potential of atom chip setups to perform metrological studies requiring high atomic mixture flux [6,33] as well as for proposed microgravity experiments that rely on the collocation of atomic mixtures [2-5]."

P5 beginning of 1st paragraph: The temperature should be quoted with the corresponding atom numbers, since atom number and temperature tend to be anti-proportional.

We thank the reviewer for this helpful suggestion. We agree that quoting the corresponding atom numbers improves the completeness and clarity of the manuscript and we have changed the paragraph accordingly as very high atomic densities could be detrimental for sub-Doppler cooling due to cooling light absorption and re-scattering.

"By subsequent optical molasses cooling, we are able to reduce the temperatures to 43 µK for $2.6 \cdot 10^7$ ^{41}K atoms and 10 µK for $9.4 \cdot 10^8$ ^{87}Rb atoms, respectively."

P5 middle of 1st paragraph: The readability would be improved by the authors also stating the size of the CMOT (even though of course it can be calculated back from the trapping frequencies).

We thank the reviewer for the suggestion to improve the readability. The initial size of the CMOT can be extracted by a time-of-flight series, that was also used to extract the temperature of the ensembles at that stage, and is measured to be 450 µm for ^{87}Rb and 570 µm for ^{41}K along the z-direction. Since the reviewer refers to P5, middle of 1st paragraph and to a back calculation from the stated trapping frequencies, we conclude that the requested size might rather refer to the initial magnetic trap, named "large volume magnetic trap" in the manuscript. We would like to note that it is not entirely straightforward to define the volume of such an anisotropic and anharmonic atom chip trap. Therefore, we would refer to the effective volume that is covered by the trapped atoms in the potential and that can be measured as the initial cloud size from in-situ measurements and back-calculated from time-of-flight series. We add this quantity, the initial magnetic trap size, to the text.

"By switching on the mesoscopic H-structure of the atom chip together with a Z-structure of the base chip, we load up to 7×10^6 (^{41}K) and 5×10^8 (^{87}Rb) atoms into a large volume magnetic trap where atoms cover an effective volume equivalent of Gaussian widths of axially $\sigma_x \approx 1200$ µm and radially $\sigma_r \approx 530$ µm at a temperature of approx. 85\,µK."

P5 middle of 1st paragraph: to the non-specialist reader it might be seen as somewhat unconventional that the author should use microwave evaporation rather than RF. They also should add a small comment as to why this is necessary.

We agree, it might be unconventional for atom chip experiments to use microwaves instead of RF. We changed the text at P5 accordingly:

"Due to the high [...] this trap permits efficient evaporative cooling of ^{87}Rb and sympathetic cooling of ^{41}K, benefiting from high thermalization rates. [...] We use microwave fields instead of a single radiofrequency to prevent the simultaneous evaporation of the ^{41}K atoms."

P7 end of 1st paragraph: "It surpasses the performance of other mobile and compact setups by an order of magnitude" This is a very bold statement, which needs citations to support it.

We have added citations to this statement linked to other mobile or compact sources of K-Rb mixtures and for planned upcoming experiments.

P7 end of last paragraph: "It follows that, if the measured relative position between the BECs Δz is zero after a fixed time of flight, the associated relative velocity has to be zero as well." This is obviously not true in the generality stated here. A single point coincidence of two trajectories might just indicate a crossing of the two rather than 100% overlap. The authors should clarify the assumptions behind their statement.

We thank the referee for this relevant comment. We agree that, in general, a single point coincidence of two trajectories does not necessarily imply a full overlap, as it may simply correspond to a crossing of the trajectories with a finite relative velocity.

In the present case, however, the two species are initially nearly overlapping at the release time t = 0. Under this assumption, and for a sufficiently long time-of-flight, a vanishing relative displacement at the detection time can be directly associated with a vanishing relative velocity along the measured direction. In the case of a non-neglectable initial shift between the two species, the zero-velocity point would be reached in the case where both species reach the same spacing as initially.

To clarify this point, the text has been modified as follows:

"Assuming the same initial position of the two species at release, if the measured relative position between the two species Δz is zero after a fixed time of flight, the corresponding relative velocity is also zero."

P9: Last paragraph*: "decaying magnetic field, which mainly acts perpendicular to the atom chip surface" The authors should elaborate why the fields are perpendicular to the surface. It seems to us that some of the physics of an atom chip relies on the fact that this is not the case (e.g. IP trap).*

We agree that decaying magnetic fields of chip traps do not necessarily lead to kicks restricted only to the perpendicular direction. However, this is approximately the case for atom chip traps based on a single/z-shaped wire + external bias field:

As illustrated in reviews on atom chip physics (R. Folman et al. *Adv. At. Mol. Opt. Phys*. **48**, 264-356, 2002), the atom chip trap forms where the circular magnetic field generated by the chip wire I_w exactly cancels the perpendicular homogeneous external bias field, B_y. The trap minimum forms at a distance $r=(\mu_0/2\pi)\, I_w/B_y$ to the chip wire, along an axis orthogonally to the chip wire and the external field vector – which is perpendicular to the chip surface (see next figure together with a sketch of the chip wire). Therefore, the movement of the trap minimum is restricted to this axis perpendicular to the surface when dynamically changing I_w or B_y. In our case, the z-shape of the wire also generates a weak trapping potential *parallel* to the chip surface (i.e. the low trap frequency). However, its zero position does not change when tuning I_w or B_y and no forces are expected to act in the direction parallel to the chip. Experimentally, this is reflected by the fact that we don't observe any displacement of the atoms parallel to the chip surface when scanning the switch-off.

This behavior is also visible in the 3D-simulated atomic trajectories during the switch-off, shown in the second plot below. It shows the trajectory during the trap release in absence of gravity. The

observed motion is essentially restricted to the z direction, confirming that the dynamics are dominated by the movement of the trap along z-direction.

In general, *additional* external bias fields can affect the zero position also along parallel directions – in this case the switch-off would be more complicated since a crossing of the trajectories in one dimension does not imply a crossing in the other. But this is not applicable to our experiment, where we only use I_w and B_y.

To clarify this in the manuscript, we add the following sentence in section 2.2:

"We point out that kicks along directions parallel to the atom chip are not observable. This is related to the condition that the position of the trap minimum depends solely on the currents through the chip wires I_{SC} and I_{BC}, as well as the external bias field B_y, and is located along an axis perpendicular to both the chip surface and the bias field [Folman2002]."

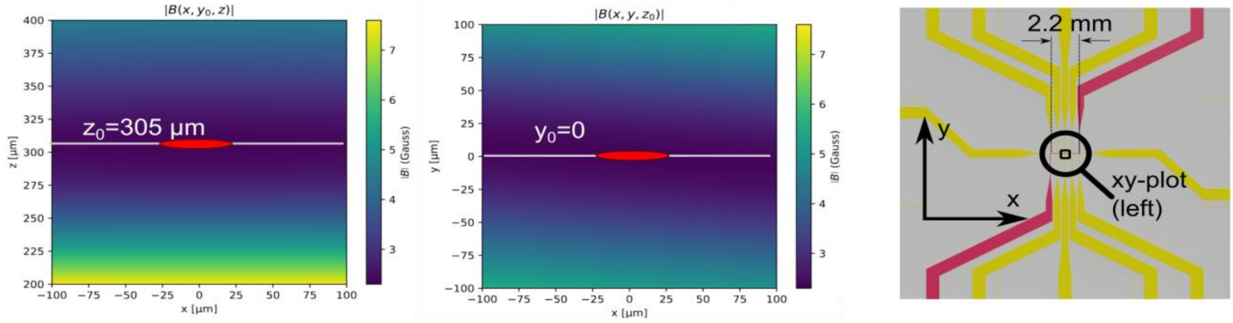

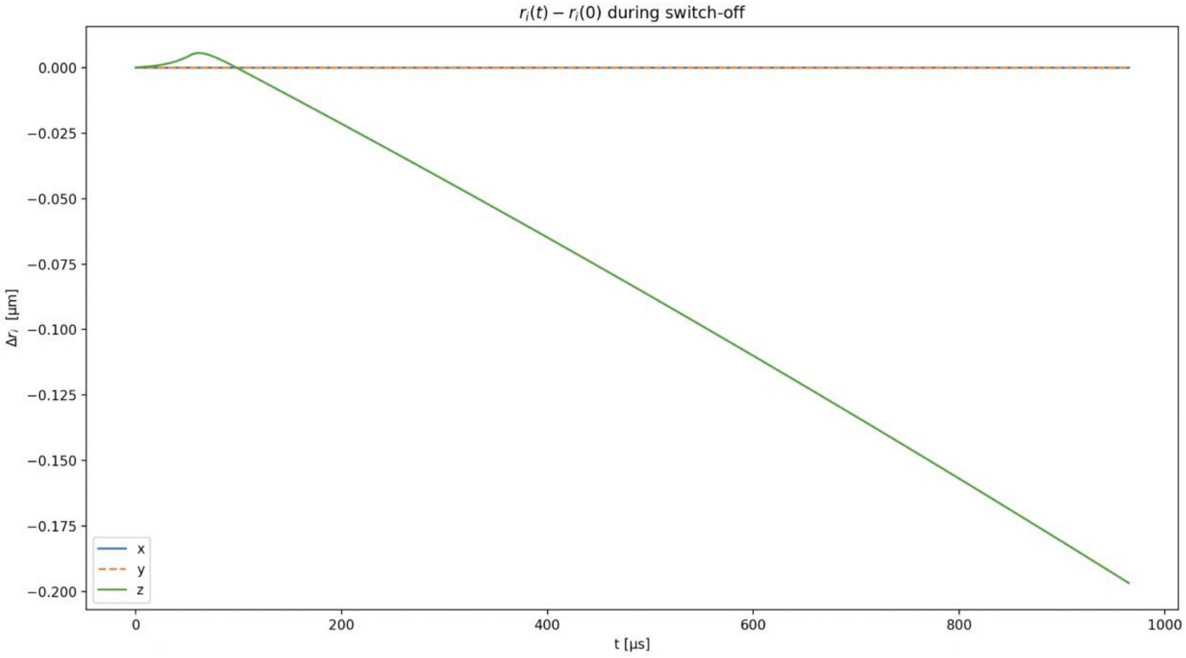

P11 second paragraph: The vertical line is derived from a single position measurement. This seems risky, since residual magnetic gradients can push the atoms towards one side, thus making them fly in an arc. Similar effects would be expected from an initial velocity. A time-of-flight measurement of multiple points would avoid such a source of error.

The reviewer makes a valid point considering the influence of magnetic field gradients on the trajectory of the atomic ensembles in free fall, and we agree that time-of-flight series could reveal such an effect. Indeed, in the process of the characterization of our experiment, we compared time-of-flight series of ^{87}Rb and ^{41}K to determine an upper bound to a residual magnetic gradient

underneath our magnetic shield of $B'_z < 0.025$ G/cm, leading to an additional relative separation of less than 5.6 µm between both species after the respective ToF which is around the position stability between the detected clouds. This impact of background magnetic fields is discussed on P12, last paragraph in the manuscript. Therefore, we expect it to be reasonable to neglect this effect in our setup and to derive the optimal atom chip switch off delay from measurements after a single ToF.

P11 last paragraph: "Given the magnitude of the different systematic effects, we attribute the observed deviations in the 0.0◦ case primarily to an oversimplified release model" This statement is troubling. In a paper, which focusses on the magnetic release, we would have expected at least some level of analysis of the systematic errors. If one direction is wrong the mere coincidence of the other two directions with the prediction does not indicate that these are right. The authors should elaborate, why they expect the 'systematic' errors only to show up in the zero-degree case.

We totally agree that the statement is a bit problematic and handwaving in this context. In the figure below, we show that the position difference between the K and Rb BECs changes significantly during the release. This would be of no effect on the GPE simulation without interactions. Considering the repulsive interactions of the two species, the interactions are enhanced if the condensates are closer to each other. As shown below, the BECs get closest in the 0° case and repel each other in the z-direction, whereas this repulsion along z reduces with the rotation towards higher angles because the condensates start to align along the y direction. Therefore, any deviation between model and experiment (which are naturally introduced by simplifications), has a larger impact on the match between GPE simulation and experiment in the 0° case than for higher rotation angles.

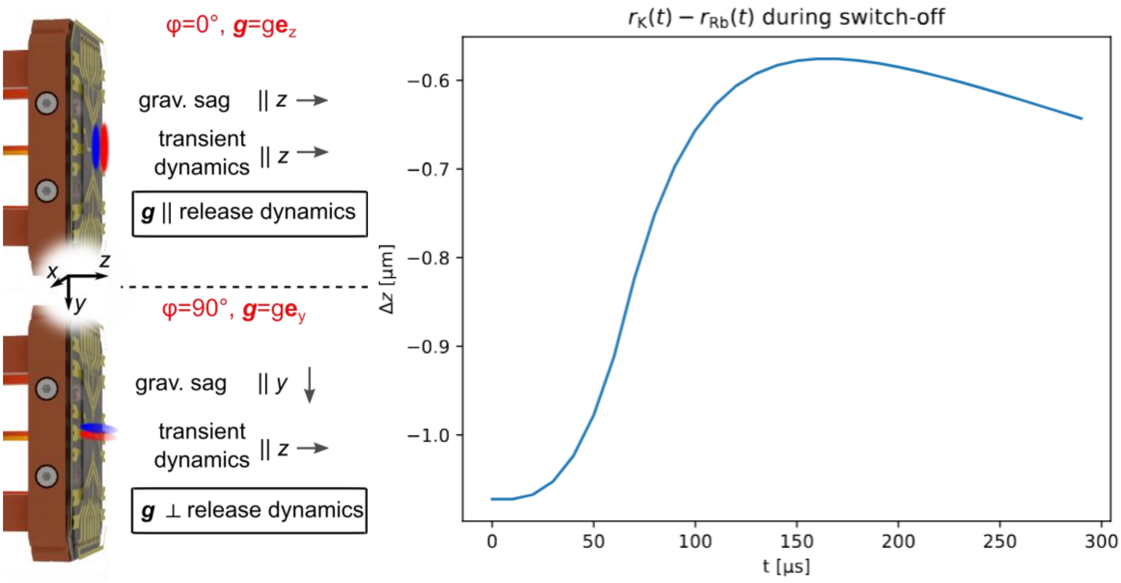

This explanation is supported by the fact that the z-position of the mixed condensates after ToF depends highly on the rotation angle of the apparatus and therefore on the orientation of gravity as can be seen in the figure below. As expected, this effect is stronger for K-41 than for Rb-87 because of its lower mass. Co-aligning gravity and the direction of the magnetic gradients during release, leads to a high displacement from K-41 (in presence of Rb-87) to the classically calculated free fall trajectory whereas rotating gravity away from the release kick direction diminishes this effect.

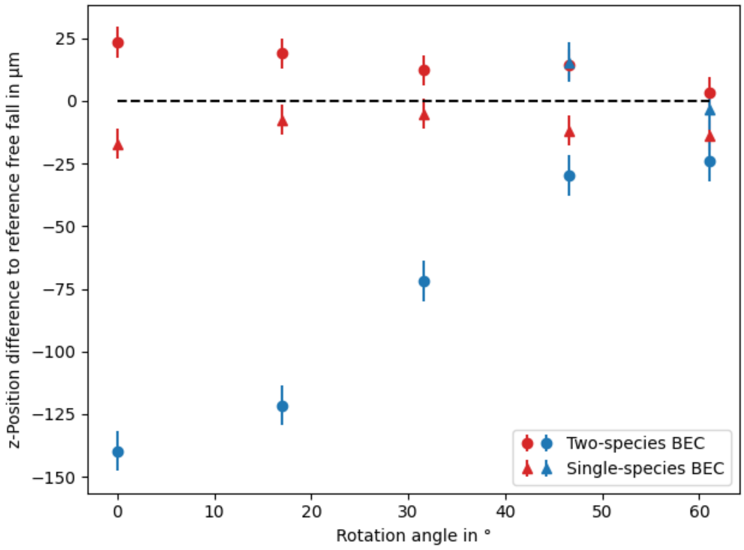

To take this into account, we add the following paragraph to the manuscript:

"Given the magnitude of the different systematic effects, we cannot attribute the observed deviations in the 0.0° case to additional background fields or atom number uncertainties. Instead, they can be explained by the trajectories of the two BECs. During release, their separation temporarily reduces along z due to transient magnetic gradients. Only in the 0.0° case, the associated atomic motion co-aligns with gravity and their mutual repulsion. This increases the interatomic repulsion compared to non-zero rotation angles. Therefore, we attribute the deviation in the 0.0° case to an oversimplified release model that does not capture the full details of the atomic motion during the transient."

P12-13: Even though conclusions are perfectly stated, a final section with the conclusions might be missing at the end of the manuscript.

We have added a final section of conclusions and outlook to the end of the manuscript:

"In conclusion, we are able to generate BEC mixtures of ^{87}Rb and ^{41}K with a high flux in different orientations to gravity as well as in microgravity. By introducing a suitable time delay between switching off the atom chip and the coils, we can tune the differential release velocity to zero, only limited by the time resolution of the hardware. The effects of gravity, interactions and transient magnetic fields are well described by a set of coupled Gross-Pitaevskii equations. Our results lay the foundation for further studies with BEC mixtures in microgravity, enabling a wide range of proposed experiments ranging from tests of the equivalence principle [6], the generation of quantum droplets [60,61] to applications in molecular physics [2,3]."

Methods, p14, figure 6: Please use physically meaningful parameters. The current in the coils are of no interest to the reader. Trapping frequencies and positions would be much more useful. Similarly, the absolute microwave frequency is of little consequence other than its detuning from the trap bottom.

We thank the reviewer for the suggestion and agree that currents are not meaningful for somebody who wants to reproduce the results, as it requires knowledge of the exact geometry of the atom chip. We have changed the following parts of Figure 6:

- Absolute microwave frequencies to frequencies relative to the non-magnetic transition |F=2, m_F=0> → |F=1, m_F=0> of ^{87}Rb.
- For the harmonic traps, we have added trap frequencies and positions.
- We have added the Mesoscopic-U/-H chip structures to give a more complete picture of the sequence (see also suggestion of Reviewer #5) including CMOT and initial magnetic trap.
- For further details we also include a reference to a PhD thesis.

6: Code availability: It is strange that the code should be available only under the condition of "reasonable request". What would be an "unreasonable request"?

We would like to point out that our code is currently available in a separate publication (see Remark of Reviewer #2 below). We also updated the Code availability statement in the paper accordingly.

Reviewer #1 (Remarks on code availability):

I cannot find any code available under the URL above.
see below

Reviewer #2 (Remarks to the Author):

I co-reviewed this manuscript with one of the reviewers who provided the listed reports. This is part of the Nature Communications initiative to facilitate training in peer review and to provide appropriate recognition for Early Career Researchers who co-review manuscripts.

We thank Reviewer #2 for the co-review.

Reviewer #2 (Remarks on code availability):

It redirects to: Efficient numerical description of the dynamics of interacting multispecies quantum gases. Annie Pichery, Matthias Meister et al. AVS Quantum Science, 5, 4, 12 2023.

I could not find any code to download, but the authors wrote that "The data that support the findings of this study are available from the corresponding author upon reasonable request".

Indeed, the reference above (Pichery et al.) does not contain the code despite being a good reference for the interpretation of the data of this paper. We made our code available in a specific publication about the numerics: "FFTArray: A Python Library for the Implementation of Discretized Multi-Dimensional Fourier Transforms, Stefan J. Seckmeyer, Christian Struckmann, Gabriel Müller, Jan-Niclas Kirsten-Siemß and Naceur Gaaloul, https://arxiv.org/abs/2508.03697v3 (2025). It is under review and expected to be published soon in "SciPost Physics Codebases". There is a section (4.5) about two-component BECs among other examples.

Reviewer #3 (Remarks to the Author):

The paper by PIEST et al. reports on K-Rb experiments on an atom chip. The experiments are carried out in the vicinity of the MAIUS-B sounding rocket payload with the prospect to be used for experiments in microgravity. However all here reported experiments have been carried out on ground, some of them were not carried out in the payload.

The main results of this work are an improvement in atom number for both species in comparison to previous chip-experiments, an optimised release method that leads to a minimum velocity difference between the two species, studies of the overlap of the two species with respect to different angles to gravity.

The described improvements are of a pure technical nature, the reported steps are incremental and largely based on standard techniques. It is not clear, why this could be seen as a scientific breakthrough.

While the work here does exceed the atomic loading rate of previous demonstrations [11,12], it does so in a ground-based setting and not under microgravity conditions, making the result somewhat less interesting to a broad audience.

We thank Reviewer #3 for the assessment of the paper. We see that mere ground-based results, even for the presented different apparatus orientations, limit the impact of the result to a more technical oriented readership. After significant efforts, we are happy to report and add recent measurements taken in the microgravity environment of the Einstein-Elevator to the manuscript. We therefore replaced two of the main figures in the paper with new ones, including microgravity results (Fig.4 and 5).

Although Fig. 2 and 3 still only show the data in the ground-based setting, these new measurements demonstrate that the apparatus is indeed able to generate BEC mixtures with similar atom numbers in microgravity. We use this new data to assess the effectiveness of our atom chip switch-off in microgravity and whether it is suitable to sufficiently reduce the release kick associated with atom chip setups.

We believe that these new results will significantly increase the impact of the paper and make it interesting to a broader readership as several groups in various international institutions propose experiments with this mixture in space and ground-based microgravity facilities.

To integrate the new data into the manuscript we have replaced Fig. 4 with an updated one and merged the new microgravity mixture result into Fig. 5. We also had to change the abstract of the paper and larger parts of sections 2.2 and 2.3 to appropriately discuss this new data.

Fig. 2 seems relatively standard - what does one learn from it?

We thank the reviewer for the comment. Although the general concept of plotting BEC fractions against each other is standard in the literature, we think that the figure provides important information that is central to our manuscript. As our apparatus is not a conventional laboratory system and can also operate in microgravity, we believe that this characterization is particularly valuable. For example, it illustrates the operational window of the apparatus for generating mixed condensates and thus provides important information for other groups considering future mixture experiments in microgravity.

Fig. 3 compares the achieved atom number and loading rate to works in literature. It is not clear why these specific references have been chosen and this should be explained better, e.g. PRL, 134, 093401 (2025) achieves a similar atom number (2x10^5 and 1.6x10^5 for Rb and K).

There was an issue with the linking of the references in Fig. 3 to the bibliography numbers, which made the references in the graph wrong. The references are now correctly/dynamically linked to the respective publications.

The apparatus used in *Phys. Rev. Lett.* **134**, 093401 (2025) (as well as other publications of the group mentioning K-Rb BEC mixtures) seems to be the same apparatus previously described in *Phys. Rev. A* **98**, 063616 (2018) in more detail. Its reported flux is thus shown in Fig. 3.

Fig. 4 shows profiles of mixed BECs after switch-off of the magnetic field in different orientations to Earth's magnetic field. The experimental data seems fairly similar for both switch-off scenarios. However experiment and theory match less well for the fast switch-off. Could the authors comment on that?

The comment refers to Fig. 5 where we have plotted the mixed BEC profiles. The *experimental data* is indeed the same for both switch-off scenarios – only the resulting s*imulated curves change*. It is thus a comparison of simulations with different assumptions rather than experiments. To better illustrate this for the reader, we have now written "Simulation with transient switch off/Simulation with immediate switch off" directly in the figure. The simulation model using the "immediate switch-off" displays a mismatch with the observed BEC positions/shapes. Only by integrating the time-constant of the decaying B-fields into the GPE simulation, the model and experimental data matches which is one of the messages of the figure.

From a technical point of view, the described work is interesting and will advance the field, the procedures are thoroughly and clearly explained and the results reported in detail. However, it is difficult to discern any major change of principle compared to previous approaches.

One of the main claims and points of interest in the manuscript is that the highest flux rate for a dual Rb-K BEC chip-system was achieved. It is not clear from the manuscript why the flux rate should exceed those previously reported and whether there is a fundamental change in the approach that leads to the improvement.

Our achieved flux does not rely on a single fundamental new principle but rather on a combination of successful techniques, including multi-layer atom chips and a combined 2D-MOT for Rb and K. The 3-layer atom chip design is key to our performance, as it combines small size with good transfer efficiency into a large volume magnetic trap and fast evaporative cooling in a microscopic, high frequency trap.

This was reflected by the sentence: "Our approach of combining a 2D-MOT and a three-layer atom chip enables us to prepare mixtures with both high flux and atom numbers.", which we change to:

"Our approach of combining a 2D-MOT and a three-layer atom chip – used for both MOT loading and evaporative cooling – allows us to achieve a high BEC mixture flux in a compact setup."

And previously in the section we have added a reference to the QUANTUS-2 experiments using the same design of a multi-layer atom chip to achieve a high Rb-87 BEC flux.

"The intermediate step of using a large volume magnetic trap to mediate the size mismatch of the CMOT to the microscopic chip trap has previously been shown in drop tower experiments [Rudolph et al., 2015] and is essential to achieve the reported flux."

We also want to point out that our novel force-free release technique to null the relative velocity of the two-species mixture has not been reported elsewhere and will be important in future chip-based mixture experiments.

In conclusion, the reported work is a relevant experimental step forwards in dual-species BEC production for space-borne experiments, but the lack of a full experimental demonstration under intended operational conditions (i.e. in microgravity) means that it is unlikely to be of the highest interest to all of Nature Communications' broad audience.

We hope that the referee would acknowledge the major efforts leading to the successful microgravity campaign. The consolidated new results are addressing their very concern, making our paper interesting for the broad audience of Nature Communications.

Minor issues:

•*Figure 2: the font size is rather small in places, especially on the axis scales, and should be increased.*

We have increased the font sizes accordingly (see also comments below for other Figures).

•*Page 5, last paragraph: the authors quote trapping frequencies for a cylindrical trap, it should be clarified whether they are radial, axial or mean.*

Throughout the paper, we have generally reported trap frequencies along all three trap axes, i.e., the eigensystem of the trap. Following the reviewer's suggestion, we now use fx and fr to denote axial and radial trap frequencies for cylindrical traps. We use fx,fy,fz or their geometric mean for more general, non-cylindrical traps. This convention has been applied consistently throughout the manuscript to improve clarity.:

(P5): "Due to the high trapping frequencies, with axial frequency $f_{x,Rb}$ = 23 Hz and radial frequency $f_{r,Rb}$ = 910 Hz for ^{87}Rb, and axial frequency $f_{x,K}$ = 33 Hz and radial frequency $f_{r,K}$ = 1326 Hz for ^{41}K, this trap permits efficient evaporative cooling of ^{87}Rb and sympathetic cooling of ^{41}K, benefiting from high thermalization rates."

(P6): "The decompressed trap has trap frequencies of $f_{x,Rb}$ = 25 Hz and $f_{r,Rb}$ = 397 Hz for ^{87}Rb, and $f_{x,K}$ = 36 Hz and $f_{r,K}$ = 578 Hz for ^{41}K."

(Fig 4): "The red curve is the expected switch-off kick in a weak magnetic trap used for atom interferometry in microgravity with eigenfrequencies $f_{Rb}={21,43,34},$ Hz and $f_{K}={31,62,50},$ Hz that is not accessible on ground."

(Fig 7): "The measurements were performed at a temperature of 100 µK and in a trap with frequencies $f_{x,Rb}$ = 23 Hz and $f_{r,Rb}$ = 910 Hz for ^{87}Rb, and $f_{x,K}$ = 33 Hz and $f_{r,K}$ = 1326 Hz for ^{41}K."

•*Most of the manuscript is well written, but in a few places the clarity could be improved. Page 9: "magnetic field prober" -> probe. Page 6 final paragraph: "For the mixture generation, only the final*

frequency of the last ramp was increased by (70±10) kHz compared to the 87Rb-only case. We account this for the few µm-shift due to the repulsion with cold 41K atoms leading to an energy shift of the 87Rb atoms." The use of "account" in this way is not, in my opinion, completely correct and the whole pair of sentences is quite clumsily structured, making it harder to read and correctly interpret.

We thank the reviewer for the suggestions and have implemented his formulations.

(P9): magnetic field prober -> probe

(P6): "For the mixture generation, only the final frequency of the last ramp is increased by (70±10) kHz compared to the 87Rb-only case. This adjustment compensates for the small spatial shift of a few micrometers caused by the repulsive interaction with the cold 41K atoms, which leads to an energy shift of the 87Rb atoms."

•*Figure 4 and discussion at top of page 9: the authors compare a theoretical model of atomic launching against experimental data. They state that "The model successfully reproduces the observed data…" but the reduced chi-squared value of 3.3 that is quoted in the caption of figure 4 is relatively high. This would suggest that either the model is incomplete or the experimental uncertainty has been underestimated. The authors should comment on what factors could be leading to the discrepancy.*

We thank referee #3 for this constructive comment and agree that a reduced chi-squared value of 3.3 indicates that the model is not fully explaining the data. Nevertheless, the qualitative dependency of the release kick felt by the atoms as a function of the switch-off's time delay is clearly understood. This measurement was based on single-point data. In the new version of the paper, we repeated this measurement: Every (black) data point in Fig4b) is the average of 10 consecutive measurements with the error bar indicating the standard deviation. The reduced chi-square to this data is now 0.94 which is close to 1.

The microgravity data matches the ground data, however due to the limited microgravity time we show only single point measurement (since every point already consists of two drops – one for Rb and one for K). The errorbars are estimated based on a separate drop campaign where we looked at the position stability of K and Rb in repeated measurements (at a shorter TOF). The reduced chi-square of the µg-model is 3.39 - mostly due to the single "off" data point at 0.08ms. We attribute the high chi-square to an underestimation of the errorbars at longer TOFs in microgravity in addition to the limited availability of further repeated measurements.

To underline this, we modified the beginning of the first paragraph in page 9 as follow:

"The higher χ^2 in microgravity is likely due to an underestimation of the positional instability of the data points, which are single-shot measurements from consecutive experimental drops."

•*Figure 4 (b): "u" should be replaced with correct character "\mu" in legend.*
Indeed. µm is now displayed correctly in the legend.

Reviewer #4 (Remarks to the Author):

I co-reviewed this manuscript with one of the reviewers who provided the listed reports. This is part of the Nature Communications initiative to facilitate training in peer review and to provide appropriate recognition for Early Career Researchers who co-review manuscripts.
We thank Reviewer #4 for the co-review.

Reviewer #5 (Remarks to the Author):

In this manuscript, the authors describe the performance of an apparatus specifically designed for fundamental physics tests to be conducted under microgravity conditions using ultracold mixtures of Rubidium and Potassium gases. Following a brief technical introduction—highlighting in particular the high atomic flux achieved through atom chip technology—the authors report on their efforts to minimize the differential velocity between the two atomic species caused by magnetic field transients during release from the magnetic trap. The final part of the paper is devoted to the study of interspecies repulsion in the presence of the Earth's gravitational field. This study is made especially interesting by the capability of rotating the apparatus with respect to the gravitational acceleration.

The manuscript is clearly written, and the information is presented in a comprehensive and well-structured manner. Regarding the content, the topics addressed are of crucial importance in view of future space missions aimed at performing fundamental physics tests with ultracold atoms. Overall, the technology and experimental solutions presented represent the current state of the art in the field. Therefore, despite the highly technical nature of the paper, I strongly recommend its publication in Nature Communications.

We sincerely thank Reviewer #5 for the positive evaluation and recommendation.

I have no major comments, only a few minor remarks:

-Figure 3: The references appear to be incorrect. For example, the reference to BECCAL is [17] but should be [16], and STE-QUEST is [7] instead of [6]. In particular, reference [12] seems not directly relevant, as it refers to an optical single-species BEC (see https://journals.aps.org/prl/abstract/10.1103/PhysRevLett.123.240402
(see above) We are sorry for the confusion. There was indeed an issue with the correct linking of the references in Fig. 3, which made the references in the graph wrong. The references are now correctly linked to the correct publications.

- Figure 6: To provide a more complete overview of the experimental sequence, I suggest adding the mesoscopic U and H structures below the atom chip.

Thank you for this suggestion. We have added the mesoscopic structures into Figure 6 to get a more complete overview of the sequence. We have also put physically meaningful numbers (like B-field strengths instead of coil currents or relative microwave frequencies) whenever it seemed possible and reasonable for us (see also comment from Reviewer #1).

- To reduce the velocity difference, the authors chose to adjust the switch-off timing between the bias coils and the atom chip. Are there physical motivations underlying this choice, or is it primarily a heuristic approach? Could the possibility of switching off the Z-base and Z-science structures at different times also play a role?
We thank referee #5 for this comment. The choice to adjust the switch-off timing between the bias coils and the atom-chip wires is related to the inductance difference between both components. As

the bias coils have a larger inductance than the wires, their switch-off will take longer than the wires one. Therefore, to keep the trap at the same position, it is necessary to compensate for this inductance difference. Considering that the Z-base and Z-science structures could in principle have slightly different inductances, adding an extra delay between their switch-off might play an additional role or offer a further degree of freedom. However, our approach is based on the large difference of inductances between Base-Chip/Science-Chip on the one side, and external coils on the other side.

- I recommend enlarging the font size of the plots in Figures 2, 4b, and especially Figure 5, as they are difficult to read when printed on A4 paper.

We have increased the font sizes of Fig. 2, Fig. 3, Fig. 4a,b. Since Fig. 5 is now even larger with the latest changes, we decided to rotate it to landscape format to make it readable when printed on A4 paper.

We sincerely thank all reviewers for their assessment and for acknowledging the substantial improvements of the last revision. We are grateful for the reviewer's thoughtful comments throughout the review process, which have helped us improve the clarity and presentation of the manuscript.

Reviewer #1 (Remarks to the Author):

The authors have substantially improved the manuscript. However, the main criticism remains: this is a rather technical paper about a incremental improvement of an experimental apparatus. No new physics and no substantially new techniques are presented. This manuscript still falls well short of the high standard of Nature Communications.

We appreciate the perspective regarding the scope and nature of the manuscript. However, we respectfully disagree with the characterization of the work as a purely technical advance. The manuscript reports both a technological advance and new physical insights enabled by the experiments. Beyond the first demonstration of BEC mixtures in a ground-based microgravity laboratory, we also show the first comparison of interacting BEC mixtures on ground and microgravity. This comparison provides, to our knowledge, the first direct experimental evidence that gravity significantly affects the ground-state configuration of an interacting quantum-gas mixture in a trapping potential.

Reviewer #2 (Remarks to the Author):

I co-reviewed this manuscript with one of the reviewers who provided the listed reports. This is part of the Nature Communications initiative to facilitate training in peer review and to provide appropriate recognition for Early Career Researchers who co-review manuscripts.

Reviewer #4 (Remarks to the Author):

I co-reviewed this manuscript with one of the reviewers who provided the listed reports. This is part of the Nature Communications initiative to facilitate training in peer review and to provide appropriate recognition for Early Career Researchers who co-review manuscripts.

Reviewer #5 (Remarks to the Author):

In this new version of the paper, the authors have significantly extended their work from both an experimental standpoint, adding a microgravity study conducted using an Einstein Elevator, and a theoretical one, providing a compelling interpretation of the discrepancy between simulations and experimental data regarding the 0° release of the condensates.

I have no specific comments and, as far as I am concerned, the paper can be published in its current form without further revisions.

Regarding the concerns raised by other referees about the technical nature of the paper, I believe the

addition of the microgravity experiment is sufficient to make the manuscript suitable for a broader readership, as the authors themselves point out. The demonstrated ability to accurately control the position and velocity of atomic samples is of crucial importance for future fundamental physics experiments in space; given the significant interest in this field, it cannot be dismissed as a mere technical detail.

We thank the reviewer for the careful evaluation of our manuscript and for the positive assessment of both the work and the revisions made during the review process.
